# Barriers and Facilitators to the Use of Wearable Robots as Assistive Devices: Qualitative Study With Older Adults and Physiotherapists

**DOI:** 10.2196/52676

**Published:** 2024-08-09

**Authors:** Leah Reicherzer, Mandy Scheermesser, Adrian Kläy, Jaime E Duarte, Eveline S Graf

**Affiliations:** 1 Institute of Physiotherapy, School of Health Sciences, ZHAW Zurich University of Applied Sciences, Winterthur, Switzerland; 2 Medbase AG, Zurich, Switzerland; 3 MyoSwiss AG, Zurich, Switzerland

**Keywords:** assistive device, barriers, facilitators, mobility, older adults, wearable robots

## Abstract

**Background:**

Light wearable robots have the potential to assist older adults with mobility impairments in daily life by compensating for age-related decline in lower extremity strength. Physiotherapists may be the first point of contact for older adults with these devices.

**Objective:**

The aims of this study were to explore views of older adults and physiotherapists on wearable robots as assistive devices for daily living and to identify the barriers and facilitators to their use.

**Methods:**

Six older adults (aged 72‐88 years) tested a wearable robot (Myosuit) and participated in semistructured interviews. A focus group with 6 physiotherapists who had a minimum of 5 years of professional experience and specialized in geriatrics was conducted. Data were analyzed using thematic qualitative text analysis.

**Results:**

Older adults perceived benefits and had positive use experiences, yet many saw no need to use the technology for themselves. Main barriers and facilitators to its use were the perception of usefulness, attitudes toward technology, ease of use, and environmental factors such as the support received. Physiotherapists named costs, reimbursement schemes, and complexity of the technology as limiting factors.

**Conclusions:**

A light wearable robot**—**the Myosuit**—**was found to be acceptable to study participants as an assistive device. Although characteristics of the technology are important, the use and acceptance by older adults heavily depend on perceived usefulness and need.

## Introduction

The maintenance of mobility is fundamental for active aging and a key determinant for quality of life in older age [1,2]. Loss of mobility in older adults occurs when the physical capacities restrict the ability to walk due to increasing age, diseases, or injuries. Aging, especially in combination with a sedentary lifestyle, leads to a decline in muscle function and cardiorespiratory fitness, which eventually results in a reduced capacity to perform daily life activities and a loss of independence [3]. This loss of autonomy caused by the decline in physical mobility presents a major psychosocial implication of aging. Adequate exercise can help mitigate these changes [3,4]. Both structured exercise and general physical activity (PA) are known to be preventive for chronic diseases, such as diabetes, stroke, osteoporosis, or obesity, and to improve mobility, quality of life, and mental health among other benefits [3]. Despite the apparent health benefit of PA [5,6], a large percentage of older adults do not meet PA guidelines in their daily lives [7]. For adults with nonreversible mobility impairment, the use of assistive technologies is considered the best option to stay active and perform activities of daily living.

These assistive technologies range from traditional mobility aids, such as wheelchairs or rollators, to powered devices such as exoskeletons. Traditional walking aids such as the rollator, while promoting mobility and facilitating leisure activities or chores such as groceries, come with disadvantages, such as being too heavy or bulky for public transport or preventing the user from walking stairs [8]. In recent years, untethered lower limb exoskeletons have emerged as wearable, robotic mobility aids that allow individuals with motor impairment to walk independently [9]. Unlike older generations of exoskeletons that present a rigid structure moving the human body, the latest wearable robots are significantly lighter and portable [10-13]. While the use of exoskeletons is mainly restricted to clinical and rehabilitation settings due to their weight, lightweight wearable robotics present a valuable alternative for private use. Therefore, they may have the potential to enable older adults to keep mobile and perform activities of daily living autonomously.

Potential benefits from the use of a wearable robot have been demonstrated by Lee et al [14], who found reduced energy expenditure and improved gait function in older adults using a soft hip assist robot. The Myosuit (MyoSwiss AG) is a recent, light wearable robot that provides users with antigravity support at the hip and the knee while standing, walking, climbing stairs, or during sit-stand transfers [15]. To date, the technology has successfully been used by people with neurological disorders such as multiple sclerosis, incomplete spinal cord injuries, or stroke. There is some evidence that activity-based training with the technology is safe, feasible, and well tolerated by patients with neurological gait disorders [16].

Experiences with other potential user groups of wearable robots as assistive devices, such as older adults, are limited. Jung and Ludden [17] found generally positive attitudes of older adults and clinicians toward the concept of exoskeleton technology but simultaneously found a reluctancy to try the technology. Shore et al [18,19] have identified several key functional requirements for designing exoskeletons for older adults, emphasizing the need for effectiveness, safety, facilitation of walking, hands-free usage, proper body support, ease of wear, and affordability to enhance their acceptance among this population. Understanding the needs and experiences of older adults and the professionals who care for them as potential user groups of wearable robots is crucial to inform future design decisions and guide implementation.

Therefore, this article explores views of older adults and physiotherapists (PTs) specialized in geriatrics on the Myosuit as an assistive device for daily living and identifies the barriers and facilitators to its use.

## Methods

This study had a descriptive design with a qualitative approach using semistructured interviews with older adults and a focus group discussion with PTs.

### Ethical Considerations

According to the federal regulations (Swiss Human Research Act, 2020), ethical approval was not required for this study. A clarification of responsibility was obtained from the Ethics Committee Zurich (No. Req-2021‐00454). Information concerning the study participation and the right to withdraw at any time was provided to all focus group and interview participants. All participants signed an informed consent form.

### The Technology

**Figure 1.**
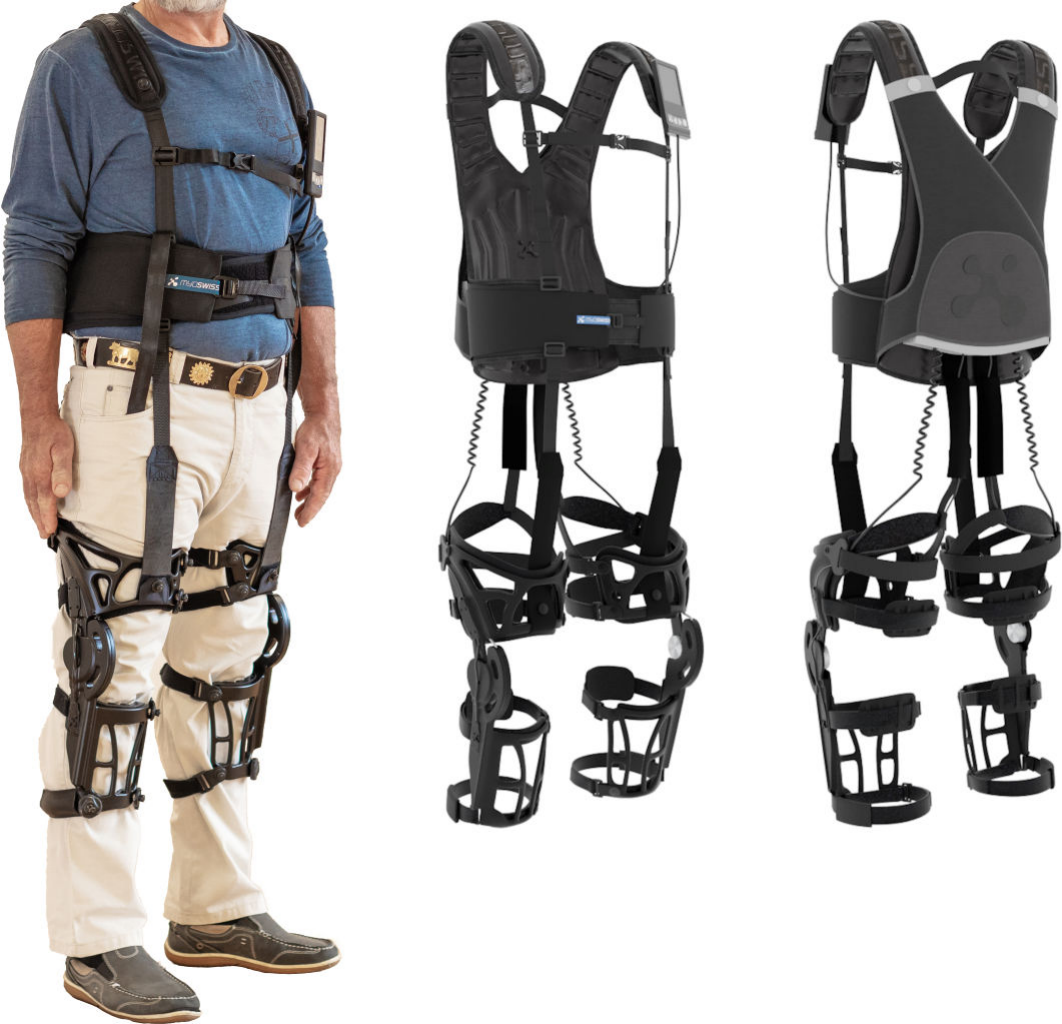
The Myosuit front and back perspective.

The Myosuit (Figure 1) is a wearable robot constructed in 3 layers that are inspired by ligaments, bones, and muscles of the human [15]. The general idea of the design is to transmit the forces over webbings and cables using different anchor points [20]. It can identify key coactivation patterns of the lower limb muscles in activities of daily life. The assistance level can be adjusted to provide forces continuously with gravity (eccentric behavior) or against gravity (concentric behavior) and for each leg individually, allowing for a high degree of personalization [21]. The current system weighs 5.56 kg including a lithium polymer battery that lasts up to 4 hours [15].

### Participants and Recruitment

For PTs, a purposive sampling technique was used for recruitment. PTs were chosen and invited via email based on their expertise in working with older adults and represented different institutions (private practices, home care, clinics, and university). Six PTs with a minimum professional experience of 5 years agreed to participate in the focus group (Table 1). Two PTs worked regularly with the Myosuit and 4 had seen or tested it but were not using it in their daily practice.

Older adults were recruited face-to-face by PTs from 2 outpatient practices between September 2021 and December 2021. The inclusion criteria for older adults in the study were age more than 65 years; ability to walk 25 m without human assistance; reduced walking speed (<0.8 m/s and >0.4 m/s); the absence of secondary neurological conditions, such as stroke; no cognitive impairment; and body height and weight in accordance with the Myosuit requirements (height: 1.5-1.95 m; weight: 45-110 kg).

Six older adults (women: n=2; men: n=4; age: mean 78.8, SD 5.7 years) agreed to test the Myosuit and take part in a first interview. Of those, 2 participants volunteered to take the Myosuit home and participate in a second interview after the 2-week trial period at home (Table 2).

**Table 1.** Characteristics of physiotherapists (PTs) participating in focus groups.

ID	Sex	Setting
PT1	F^a^	Geriatric inpatient clinic
PT2	F	Geriatric inpatient clinic and university
PT3	F	Acute inpatient and outpatient setting
PT4	F	Neurological outpatient rehabilitation
PT5	F	Geriatric outpatient clinic
PT6	M^b^	Home care

a F: female.

b M: male.

**Table 2.** Characteristics of older adults (participants [P]).

P	Sex	Age (years)	Living situation	Interview 1	Interview 2
P1	M^a^	88	Alone, with support	✓	
P2	F^b^	72	With spouse	✓	
P3	F	77	Alone, with support	✓	✓
P4	M	85	With spouse	✓	
P5	M	75	Alone, no support needed	✓	✓
P6	M	76	Alone, no support needed	✓	

a M: male.

b F: female.

### Data Collection

One web-based focus group with PTs (n=6), who specialized in geriatric care, was conducted to capture professionals’ views on the technology. A semistructured topic guide was developed according to Benighaus and Benighaus [22] by the interdisciplinary team, involving a movement scientist (ESG), physiotherapist (LR), and social scientist (MS) experienced in qualitative data collection and usability or user experience research. The focus group was moderated by MS with LR present for note-taking and recording. Discussion topics revolved around the experts’ opinions on a wearable robot as an assistive device for older adults and which barriers and facilitators they anticipate from a professional point of view. The duration of the focus group was 1.5 hours. The audio recording of the web-based discussion was transcribed verbatim.

Older adults (n=6) were invited to try the Myosuit in a session with a physiotherapist (AK) and a physiotherapy research associate (LR), followed by a semistructured interview. The data collection took place at the participants’ local physiotherapy practice or in suitable rooms at the university campus.

Before testing the Myosuit, the participants were informed about the study procedure and goals. Subsequently, written informed consent was obtained. Participants were introduced to the Myosuit in several steps: (1) a short explanation of the functions and purpose of the device, (2) individual adjustment of the straps and backpack to the participant, and (3) performance of a set of easy tasks with assistance of the Myosuit. These tasks included transferring weight from one leg to the other; standing up from a chair; and, once the participants felt confident, walking and stair climbing up and down using their habitual walking aid. The participants were encouraged to take the Myosuit off by themselves and put it back on. The introduction was video recorded.

**Figure 2.**
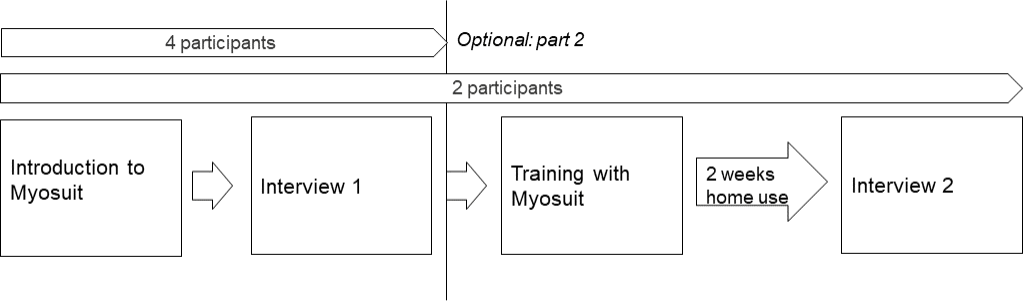
Data collection procedure for interviews with older adults.

Following the introduction, semistructured interviews were conducted by one of the authors (AK) in German. First, demographic information, such as age, gender, and living situation, was discussed, followed by general interest in technology and perception of individual health status and abilities. The main part of the interview focused on first impressions of the technology and the perceived barriers and facilitators to using the Myosuit in daily life. The topic guide (Multimedia Appendix 1) was developed by the interdisciplinary research team. The questions were informed by the theoretical domains framework [23,24], which provides a comprehensive, theory-informed approach to identify personal and environmental influences on a behavior (here: use of the wearable robot). For example, to address social influences, we included the following question: “How would you imagine your friends and family react to you using this technology?” A pilot test with 1 older adult was done to test the topic guide before conducting the interviews and to assess whether the steps for introducing the technology were feasible. Interviews lasted between 30 and 45 minutes and were audio recorded. Participants who volunteered to take the Myosuit home (n=2) received another training session with the Myosuit supervised by a physiotherapist (AK) at the beginning of a 2-week period (Figure 2). Another visit was scheduled after 1 week in case the participants had questions about the use of the Myosuit. A second interview was conducted at the end of the 2 weeks of using the Myosuit at home. Audio recordings from all interviews and participants’ statements from the video recordings were transcribed verbatim.

### Data Analysis

Two researchers (LR, MS) coded the transcripts of interviews from both time points and focus groups independently for a thematic qualitative text analysis according to Kuckartz [25], using the software ATLAS.ti (Windows Version 9.1.7). This approach allows for the thematic analysis of different types of interviews, as well as other types of data, such as focus groups [25]. The analysis process involves familiarization with the textual data through repeated reading, highlighting of important passages, identification of codes, and synthesis in larger thematic categories. The development of main topical categories was guided by the interview guidelines, but inductive analysis of unanticipated topics or meanings was also considered. After a first round of coding using the main categories, the initial categories were discussed and combined where deemed appropriate, and subcategories were determined. The category system was reviewed, discussed, and adapted until deemed comprehensive. The final category system comprised 8 main and 17 subcategories (Multimedia Appendix 2).

Once all data were coded using the final category system, the information with related meanings across interviews and focus groups was summarized, and redundant information was reduced. Finally, the categories were analyzed, and the content was organized into barriers and facilitators of using the Myosuit as an assistive device, considering factors related to the technology, the individual, and the environment.

## Results

The results are presented for the main categories. Quotes and pseudonyms (“P” for the participating older adults and “PT” for physiotherapist) are used in the following sections to illustrate the categories.

### The Technology

Regarding the technology, various usability aspects, such as the process of donning and doffing, the comfort, or sound, were identified as factors that may influence the use.

### Usability of the Technology

While using the technology for the first time, the majority of participants felt that the initial donning and doffing was not as easy as they had imagined it but were also under the impression that they could don and doff independently: “It [handling] is quite good. You have to get used to it of course. But it’s positive” (P5). PTs also considered independent donning and doffing as a challenge for older adults and were under the impression that most older adults would require assistance by family or care providers. One participant who had seen a video of the technology previously was under the impression that it had appeared to be easier to use in the video. More specifically, participants described an initial overwhelming feeling concerning the straps that need to be fixated at the right place on the user’s body: “all these straps and I have no idea which one goes where” (P1). Enough hand strength was mentioned as a requirement:

*You would have to think about making the buckles on the legs so that they are easier to click into place. Because for people with weak hands it can be quite difficult, because it also has to fit tightly*.

[P3]

PTs brought up the comfort of wearing the technology, especially for extended periods of time, which would be necessary for an assistive device: “there is pressure on it when you wear it for a long time. And if you are sensitive to it now, it can be painful” (PT4). Indeed, a few participants noted that the force application does feel uncomfortable at times: “The settings sometimes are more comfortable or a bit more uncomfortable when it like jams or rumbles on the back” (P3). Three participants felt restricted in their mobility by the technology rather than feeling like it supported their movement, especially on the stairs. One user attributes this to the weight of the technology. One PT provided another explanation: she had observed that her patients who have more pronounced gait deviations initially struggle with the gait pattern of the Myosuit that supports hip and knee extension. For some participants, the technology felt rather heavy at first but was not as noticeable once the hip belt was properly attached.

Navigating the control unit and the manual selection of the appropriate modes (ie, concentric or isometric) were brought up by the experts as a potential difficulty to anticipate. However, the user interface was generally received positively by older adults. One participant made a statement that she would need practice to navigate the user interface and to train with someone who is experienced with the Myosuit.

*It gets quite complicated; you have to be sure, but you also need to practice multiple times with someone who knows how to do it.*

[P3]

After regular use, however, the display did not pose a challenge anymore.

Many participants observed and mentioned the sound the technology makes. For some, the sound was too loud: “Yes, maybe just the sound it makes. If I were to go for a walk with someone, if I were to do that, I wouldn’t find it so pleasant” (P6). Participant who home tested noted that the sound is not as noticeable when using it outside as compared with indoors:

*Yes, well, I can live with the sound now. Outside you don’t notice it so much. Because I walk next to the streets where there is a lot of noise anyway. You don’t hear it there.*

[P3]

### The Individual

On the level of the individual, the general attitudes toward technology, fear of falling, and individual walking capabilities, as well as the expected and perceived benefits of using the technology, were identified as barriers or facilitators.

### Attitude Toward Technology

Technology acceptance by either the older adults themselves or the therapists as one important point of contact with such technologies was identified as a potential barrier by PTs. On the contrary, most older adults in this study expressed that they were open and interested in new technologies. Digital media are part of their everyday lives, and they use digital technologies to measure their daily activity, such as pedometers or fitness trackers. One participant (P3) commented, “Of course not [only] for health,…I have a laptop and do most things online.”

### Fear of Falling

Participants described how they are afraid of falling in everyday life. One of the participants who decided to test the technology at home has had several falls without injuries previously. She described using a walking stick in combination with the Myosuit, which made her feel safer. For some participants, donning and doffing, the weight, or the force application caused a fear of falling:

*The backpack is bothering me. Also, because it is pulling me backwards and makes me feel insecure that I might fall over. And I do not want to fall*.

[P1]

PTs also considered whether fall risk might be a potential barrier to its use:

*I also thought about individuals with gait instability. Whether there is experience in that area [with the Myosuit] and whether it might even be more hindering and possibly contribute to the risk of falls*.

[PT3]

### Individual Walking Capabilities

Most participants were capable of independent walking, with walking durations ranging between 10 minutes and 2 hours (long walks). Five participants were walking with walking sticks or hiking poles. One participant had no walking aid at all. Participants who described higher individual walking capabilities tended to be less interested in Myosuit:

*I prefer to walk the stairs myself. I prefer to go for a walk or hike myself. I much prefer to exercise a little in the studio or in physiotherapy. I imagined it [a wearable robot] very differently*.

[P1]

### Expected Benefits

Participants hoped for various immediate or long-term results from using the Myosuit, which can be summarized as expected benefits. These expected benefits were the main motivators for participants to test the Myosuit. One expected benefit was to increase mobility outdoors without depending on aids such as a wheelchair “to be able to go outside and not sit at home.” One participant said:

If the walking sticks might not be sufficient anymore. Where I live, I see many people using walkers and that is not for me. And wheelchairs even less, that would be my very last option. Anything that allows me to stay mobile independently is positive for me.

[P3]

PTs specialized in geriatric care similarly voiced openness toward using a wearable robot as an assistive device if it would help their patients maintain or improve independent mobility. Some older adults expected to see effects like an improved walking ability or balance: “That was the main reason, I wanted to try the technology. Whether it helps to improve my walking” (P1). One participant was under the impression that walking with the Myosuit could be more fun and therefore increase walking distance: “Possibly, maybe I would walk to [destination in town] twice more than right now. It could be that I would have more fun then, that’s quite possible” (P6).

### Perceptions of Benefit

Most respondents addressed the perception of benefit, reflecting the positive outcomes they experienced as a result of using the Myosuit. The range of perceived benefits spans from “more safety” and “more mobility” to “realizing one’s own goals.” One participant (P3) did not expect much from the technology and was then pleasantly surprised. This participant described the following: “I just felt safer than if I had gone without.” Consequently, their mobility increased: “Especially to go for more walks. I was practically out every day except yesterday and the day before yesterday…. But one day, I think I even managed 2400 steps.” The participant also noted that not only did the intensity of the movement increase but the quality of the movement also changed: “Well, I was able to take longer steps and I walked faster” (P3).

Others saw no personal benefit in the wearable robot for themselves or perceived a discrepancy between the benefits they had expected and their actual experiences. “…on the video on the internet, the enthusiasm was really great…. One even did a marathon…. But I don’t see that at all. The support is not enough so that I could do that” (P4).

### The Environment

The environmental factors identified in this study include appropriate use situations, social influences, and costs associated with the introduction of a novel technology.

### Use Situations

Participants were asked to describe contexts or scenarios in which they could envision themselves using the technology. They primarily imagined using the Myosuit for walking activities outdoors, or potentially for tasks such as groceries or day activities. PTs discussed that home use would be more beneficial than using it during a therapy session, stating, “If it could be managed with home care services or with family members, and simply say, ‘He wears the suit for two or three hours a day, once a day, and tries to manage everyday life.” It became clear that participants also preferred to remain within the closer surroundings of the home. One participant described not wanting to use the Myosuit for activities with longer duration that would require boarding a train and take her further away from home: “So in everyday life I used it to walk more. I didn’t dare to go to the city with it with the trains and trams…” (P3).

### Social Influences

Family support, as an import prerequisite to putting on the Myosuit (P1) or in motivating people to try out new technologies (P4), was reported by the participants. PTs anticipated reluctance from older adults to use assistive technologies that are associated with older age:

*I’m already struggling to convince some residents in the facility to use a walker because they think, ‘I’m not old.’ They believe that walkers are for the elderly, and we’re talking about people who are over 80 years old*.

[PT2]

PTs also considered social desirability and were unsure whether older adults would consider wearing the Myosuit in public:

*After all, you look different and if you need it in everyday life and you have this thing on, you have to be confident enough to answer questions from those around you*.

[PT4]

Worries about how this type of wearable robot is perceived by others were also expressed by older adults regarding the sound and looks of the Myosuit: “Of course, if I go out on the street now, someone will be looking. But I am so self-confident in my age that it doesn’t bother me” (P3). Some participants were hesitant to wear the Myosuit outdoors: “I wouldn’t have…the guts yet to go to a supermarket with it.… I don’t think so…” (P6).

### Costs

Reimbursement schemes in the health care system were discussed as a barrier for use of the technology in daily clinical practice by PTs, as usually 1 session per week is reimbursed by health care insurances and this time is often too short for PTs to implement new technologies. It was important for participants that the benefit outweighs the costs:

*It’s…certainly worth the price if I think I that I could walk a little better in everyday life and take a few steps with someone*.

[P6]

## Discussion

### Principal Findings

**Figure 3.**
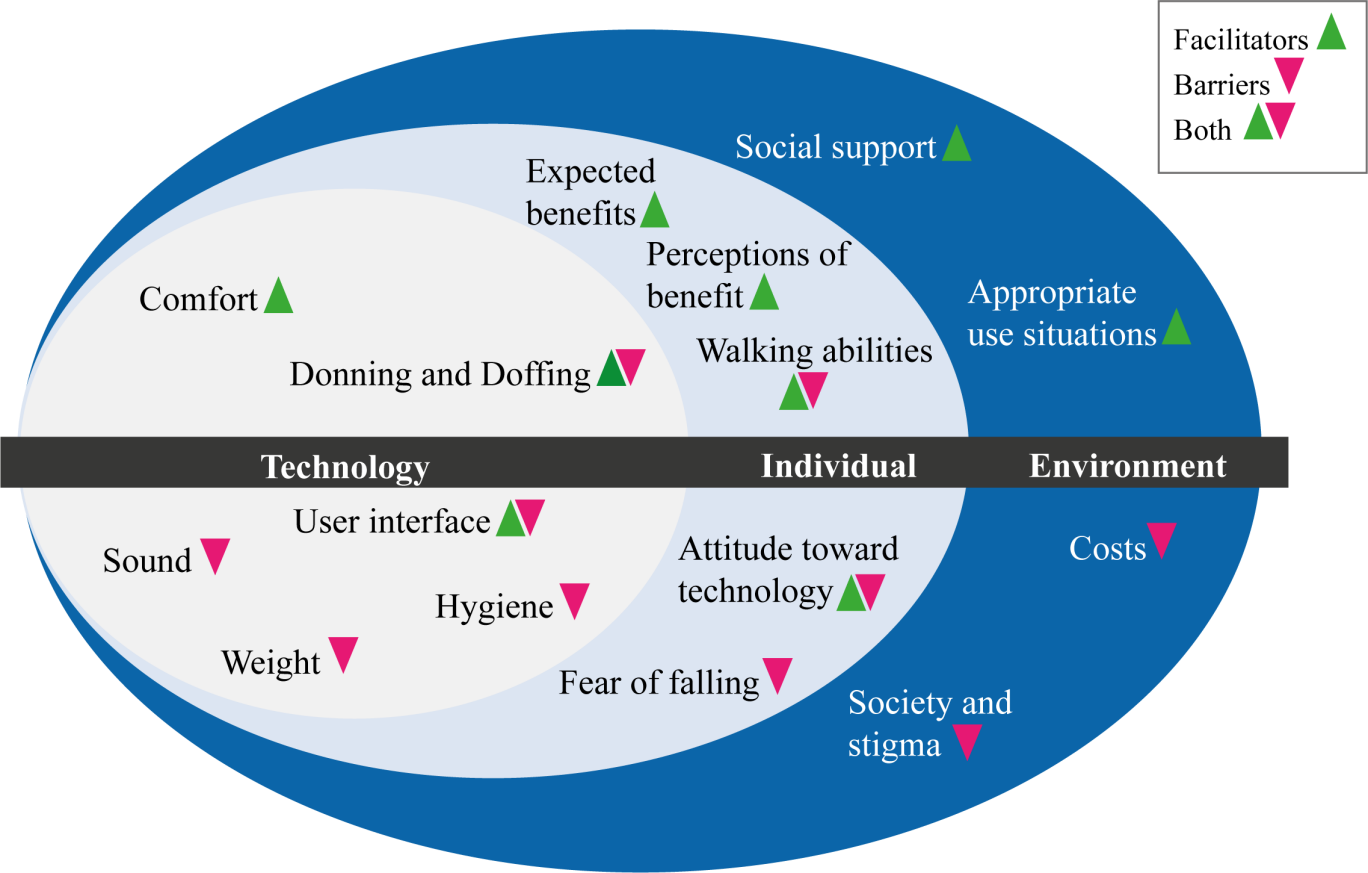
Barriers and facilitators to the use of the soft exoskeleton identified by older adults and physiotherapists.

The results demonstrate a generally favorable attitude among older adults and PTs toward the novel wearable robot. Several participants described how they experienced greater stability while standing, walking, and climbing stairs or during the sit-to-stand transfer. The technology made movement easier, and noticeable support was mentioned. Two older adults volunteered to test the Myosuit at home for an extended period and used it as a support during daily life activities. They reported an increase in walking distance and in general motivation for PA. Most other participants, however, did not see the need to use this type of technology. Main barriers to its use were factors centered around the individual (eg, the perception of “not needing it” or attitudes toward wearable robots), the technology (eg, ease of use), and the environment (eg, the support received; Figure 3). These results are in line with previous literature on factors influencing acceptance of new technologies. A systematic review [26] identified concerns regarding the technology (eg, costs), expected benefits of technology (eg, perceived usefulness), need for technology, alternatives to technology, social influence, and characteristics of older adults (eg, desire to age in place) as factors influencing the acceptance of technology in a preimplementation stage.

Contrary to common beliefs, previous studies [27,28] did not find a significant association of age with acceptance or general attitude toward robots. The adoption of technologies in the older age group has indeed grown considerably in the last decade, but a substantial gap remains between younger and older adults [29]. It has been demonstrated that preconceptions of older adults about robots are ambivalent, and while they may be open to the idea, they are not prepared to actually use them [30,31]. Frennert and colleagues [30] described a tension between seeing the benefits of a robot and simultaneously having the attitude of “good for others but not themselves.” Similarly, participants in this study were under the impression that they themselves do not need such technology. This may indicate that the social stigma pertaining to assistive technology of users being old or disabled also extends to these novel assistive devices. It further raises the question how best to determine who may benefit from this technology based on indicators of functional capacity rather than pathology. We composed our sample of older adults with measurable reduced walking speed. However, several participants did not feel limited in their mobility in daily life and did not benefit from using a wearable robot. A combination of functional mobility tests and self-reported mobility assessments to identify who may benefit seems like a more promising strategy.

Perceived usefulness of the technology was identified here as another central influence on acceptance (intention to use) of the technology. If a participant did not see a benefit or value when first trying the wearable robot, it was unlikely that it was given a second chance. Previous studies of assistive technologies for older adults identified added value as a central facilitator to technology use in general [32-34]. Similarly, it is apparent in numerous studies that users’ perceptions of assistive wearable robots are influenced by the experience of using the technology and that adoption of the technology is dependent on users regarding them as valuable for their own purposes [35]. By compensating for diminished mobility and enhancing exercise tolerance, wearable robots are uniquely positioned to be used by older adults for increased total PA, exercise, and social interaction [36]. Chen et al [37] have studied older adults’ intention to use exoskeletons and highlight that practitioners should focus on encouraging favorable attitudes and perceptions toward robotic technologies by communicating the benefits and value that wearable robotics can provide to potential users.

Fewer studies to date have investigated the reasons behind nonuptake and uptake of technologies by PTs. Systematic reviews in the field of digital health have found that complexity, costs [38], and lack of reimbursement [39] act as barriers to the implementation of telehealth. In our study, PTs were more critical regarding the usability and expressed concerns about the complexity of the technology. They suspected that older adults would encounter difficulties with the control unit or with donning and doffing at home, which was expressed less frequently by older adults themselves. This may reflect not only a general tendency of Western societies to be more conservative with regard to technological devices than other societies [40] but also a tendency of health care providers. In addition, there is currently no reimbursement scheme that factors in the time needed to successfully implement a new technology into a therapy setting, presenting a considerable barrier for PTs to adopt new technologies [41]. This should be addressed, as PTs are in a unique position to introduce their patients to novel technologies that can foster their autonomy in daily life.

It was universal across interview and focus group participants to emphasize the importance of ease of use of the technology and an inconspicuous appearance while being comfortable. A previous qualitative study [42] similarly identified the device appearance and comfort as important, with a discreet color and materials with a comfortable feel being favored by adults with mobility impairments of different origin.

This further illustrates that while it is important to meet older adults’ needs by providing the expected benefits, it is equally important that technology is easy to use, in order for these benefits to be realized [43]. Technologies that are designed without considering the specific user group’s needs and preferences are less likely to be used. Future design iterations may therefore focus on comfort, the simplicity of donning and doffing, and the user interface for intuitive use.

### Limitations

It should be noted that the participants most likely had a favorable attitude when approaching the study, presumably because their participation was linked to curiosity about robot-assisted training or expectations regarding the benefits of the technology. Testing the device in the home environment or therapy setting likely allowed older adults to develop a good understanding of barriers and facilitators to the use of wearable robots. Data saturation was likely not reached with the conducted interviews, as we had to base our sample on availability. This may limit the informative value of the results. However, triangulation was used by combining 2 different data collection methods and including different user groups to enhance the breadth of information. Face-to-face focus groups would have been well suited for this purpose, as they allow for personal contact between the interviewer and the PTs. Web-based focus groups, on the other hand, were less suitable but were considered necessary due to the limitations of the COVID-19 pandemic.

### Conclusions

This article provides valuable insights into the barriers and facilitators influencing the use of a novel wearable robot from the perspective of older adults and PTs. The results indicate a generally positive attitude toward the technology and highlight the importance of perceived usefulness and value besides the specific characteristics of the technology to realize its benefits.

To overcome the barriers and capitalize on facilitators, the following points should be considered for future action. First, there is a need to clearly communicate the potential benefits and value of the technology, emphasizing how it can address specific challenges faced by older adults and enhance mobility. Second, ease of use should be prioritized through intuitive interfaces and straightforward controls to facilitate integration into daily life activities. Third, providing adequate support, including clear instructions and resources, is crucial to ensure successful adoption and use of the wearable robot.

## Supplementary material

10.2196/52676 Multimedia Appendix 1 Interview questions.

10.2196/52676 Multimedia Appendix 2 Category system.

## Abbreviations

PA: physical activity
PT: physiotherapist
