# Supplementary material for: Barriers and Facilitators to the Use of Wearable Robots as Assistive Devices: Qualitative Study With Older Adults and Physiotherapists
Source: JMIR Rehabil Assist Technol. 2024 Aug 9;11:e52676. doi: 10.2196/52676 (PMC11327840; doi:10.2196/52676)
Supplement: Multimedia Appendix 1 [file rehab-v11-e52676-s001.docx]

**Appendix 1. Interview questions**

- What would you use the Myosuit for?
- What would be reasons for you to use the Myosuit?
- What advantages could using the Myosuit have? What disadvantages could using the Myosuit have?
- How do you feel about trying the Myosuit alone? What do you do about any concerns? What reinforces you? How confident do you feel using the Myosuit?
- What do you think, could you imagine using the Myosuit alone/with your partner? What support would you need to use the Myosuit?
- What expectations did you have for the Myosuit?
- What do you think will happen if you tell your family/friends that you are using the Myosuit?

**Conclusion**

- Is there anything I haven't asked yet but you would like to add?

**Interview 2**

- How have you been feeling in the past two weeks? Physically? Psychologically?
- Tell me a bit about how it was to use the Myosuit at home?
- Do you think the Myosuit has an impact on your health or physical activity? In what way?

**Myosuit**

- Has your initial impression been changed/confirmed? In what way?
- What do you think was done well, and what could have been improved?
- Where have you used the Myosuit? Do you have any additional ideas for where you could use the Myosuit?
- How did you feel about trying the Myosuit alone? How did it feel to use the Myosuit alone?
- What do you do to address any concerns? What strengthens you? How confident do you feel about using the Myosuit?
- What additional support would you need to continue using the Myosuit?
- How did it feel to use the Myosuit in public?
- To what extent were your expectations of the Myosuit met/not met? Improvement/deterioration of your daily activity?

**Conclusion**

- Is there anything we have not discussed that you would like to add?
