# Supplementary material for: Barriers and Facilitators to the Use of Wearable Robots as Assistive Devices: Qualitative Study With Older Adults and Physiotherapists
Source: JMIR Rehabil Assist Technol. 2024 Aug 9;11:e52676. doi: 10.2196/52676 (PMC11327840; doi:10.2196/52676)
Supplement: Multimedia Appendix 2 [file rehab-v11-e52676-s002.docx]

**Appendix 2. Category System**

| **Main category** | **Subcategory** |
| --- | --- |
| **The individual** | |
| **Attitude towards technology** | - |
| **Concerns: Fear of falling** | - |
| **Individual walking capabilities** | - |
| **Perceptions of benefit** | Feeling safer |
|  | Reduced pain |
|  | Increased Mobility |
|  | Increased Stability |
| **Expected benefits** | Improve or maintain body function |
|  | Mobility and independence |
| **The environment** | |
| **Use situations** | Daily life activities |
|  | Walking |
| **Social influences** | Support and encouragement by family or caregivers |
|  | Society and Stigma |
| **The technology** | |
| **Usability of the technology** | Complexity of user interface |
|  | Battery life |
|  | Belt and straps |
|  | Overall comfort |
|  | Sound of the motor unit |
|  | Unfamiliar movement and feeling of the body |
|  | Weight |
